# Supplementary material for: Age-Dependent Contribution of Domain-General Networks to Semantic Cognition
Source: Cereb Cortex. 2021 Aug 31;32(4):870–90. doi: 10.1093/cercor/bhab252 (PMC8841593; doi:10.1093/cercor/bhab252)
Supplement: SupplementaryMaterial_MDN_LANG_bhab252 [file supplementarymaterial_mdn_lang_bhab252.pdf]

Age-dependent contribution of domain-general networks to semantic cognition

–

Supplementary Material

Materials and Methods

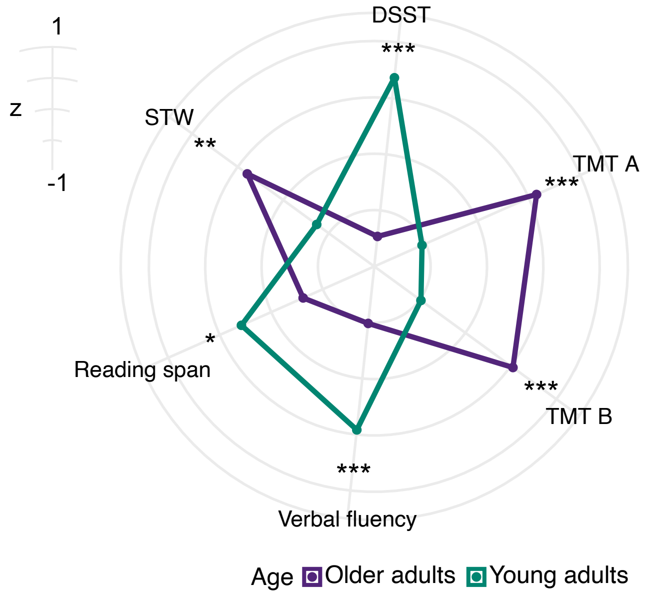

Supplementary Figure S1. Age differences in neuropsychological tests. STW = Spot-the-word test, DSST = Digit symbol substitution test, TMT A/B = Trail making test A/B. *** $p < 0.001$, ** $p < 0.01$, * $p < 0.05$.

Data Analysis

Table S1. Age-specific regions of interest (ROIs) within domain-general networks.

ROI	Hemi	*x*	*y*	*z*	Region
**Older adults**					
**MDN (from contrast Semantic fluency > Counting)**					
1	L	-9	15	51	Pre-SMA
2	L	-31	25	4	Insula
3	R	31	27	2	Insula
4	L	-34	0	57	MFGd
5	L	-44	5	35	MFGv
6	R	43	35	32	MFG
7	L	-14	-65	51	SPL
8	L	-11	-72	10	IntraCAL
9	R	18	-80	7	IntraCAL
**DMN (from contrast Counting > Semantic fluency)**					
10	R	6	-52	38	Precuneus
11	R	51	12	-31	TP
**Young adults**					
**MDN (from contrast Semantic fluency > Counting)**					
1	L	-4	2	29	dACC
2	L	-31	25	2	Insula
3	R	31	27	2	Insula
4	R	36	42	32	MFG
5	L	-29	-65	51	SPL
**DMN (from contrast Counting > Semantic fluency)**					
6	L	-56	2	-20	MTG
7	R	8	-65	29	Precuneus
8	R	51	10	-31	TP

Coordinates are given in MNI standard space. Abbreviations: Hemi Hemisphere; Pre-SMA Pre-supplementary motor area; MFG/MFGd/MFGv Middle frontal gyrus dorsal/ventral; SPL Superior parietal lobe; IntraCAL Intracalcarine gyrus; TP Temporal pole; dACC Dorsal anterior cingulate cortex; MTG Middle temporal gyrus; MDN Multiple-demand network; DMN Default mode network.

Head motion and functional connectivity

We calculated the following analyses to further ensure that our results of the functional connectivity analyses were not confounded by head motion.

Firstly, we checked whether head motion was correlated with our functional connectivity measures. To this end, we calculated the root mean square (RMS) of realignment parameters for each participant (Power et al., 2014) and Pearson correlations for each functional connectivity measure and age group. Results did not reveal any significant correlation for within-MDN functional connectivity (OA: $r = 0.051$, $p = 0.8$; YA: $r = 0.032$, $p = 0.87$), within-DMN functional connectivity (OA: $r = 0.058$, $p = 0.77$; YA: $r = 0.062$, $p = 0.75$), and between-network functional connectivity (OA: $r = 0.03$, $p = 0.88$; YA: $r = 0.087$, $p = 0.65$). The script

Secondly, to further rule out any potential impact of head motion on our results, we performed the following supplementary statistical analyses with motion RMS being added to the GLM as a covariate. The key statistical conclusions remain the same. (i) For within- and between-network functional connectivity (equivalent to statistical analyses for figure 7B), we found significant effects for within-MDN ($\beta = 0.07$, $t = 2.01$, $p = 0.049$) and between MDN and DMN connectivity ($\beta = 0.12$, $t = 2.88$, $p = 0.006$) which confirms our previous results. As before, there was no difference in strength of functional connectivity between age groups (all $p > 0.3$). Further, there was no effect of motion RMS on any within- and between-network connectivity (all $p > 0.68$). (ii) For the effect of functional connectivity on task performance (equivalent to statistical analyses for figure 7C), we performed mixed-effects models with motion RMS as an additional covariate. For accuracy, we found a significant interaction between age and functional connectivity between MD and DM regions ($\chi^2 = 4.64$, $p = 0.03$) which was not detected previously. Results showed that older adults' accuracy during semantic fluency decreased with strengthening functional connectivity between networks, while young adults showed the opposite pattern (Fig. 1). This finding underlines our interpretation of an age-dependent efficiency of task-relevant networks in semantic cognition.

For response time data, results did not differ with respect to the statistical models that did not include a covariate for motion RMS. We found significant interactions between age and all functional connectivity measures: within-MDN functional connectivity ($\chi^2 = 32.29$, $p < 0.001$), within-DMN functional connectivity ($\chi^2 = 35.55$, $p < 0.001$), and between-network functional connectivity ($\chi^2 = 21.18$, $p < 0.001$).

Supplementary Results

Behavioral Result Tables

Regression tables were generated using RStudio (R Core Team, 2018) and the package sjPlot (Lüdecke, 2020).

Table S2. Results for mixed-effects models for accuracy and response time.

	Accuracy			Response time		
Coefficient	*Log-Odds*	*Conf. Int (95%)*	*p*	*Estimates*	*Conf. Int (95%)*	*p*
Intercept	6.28	5.21 – 7.34	**< 0.001**	6.45	6.41 – 6.49	**< 0.001**
Age	-3.94	-5.96 – -1.92	0.136	0.01	0.00 – 0.02	0.072
Condition	-6.47	-8.58 – -4.36	**< 0.001**	0.17	0.13 – 0.22	**< 0.001**
Difficulty	4.63	2.53 – 6.73	**< 0.001**	-0.06	-0.11 – -0.01	**< 0.001**
Education	-0.11	-0.24 – 0.01	0.082	-0.01	-0.01 – 0.02	0.058
Age * Condition	7.26	3.23 – 11.29	0.162	0.08	0.06 – 0.09	**< 0.001**
Age * Difficulty	-7.99	-11.95 – -4.03	**0.002**	0.00	-0.01 – 0.02	0.7
Condition * Difficulty	-5.03	-9.22 – -0.84	0.049	-0.10	-0.20 – -0.00	0.056
Age * Condition * Difficulty	14.95	7.05 – 22.84	**0.002**	0.03	-0.00 – 0.07	0.092
**Random Effects**						
σ^2	3.29			0.10		
τ_{00}	0.19 $_{Subj}$			0.01 $_{Subj}$		
	0.22 $_{Category}$			0.00 $_{Category}$		
ICC	0.11			0.08		
N	58 $_{Subj}$			58 $_{Subj}$		
	22 $_{Category}$			22 $_{Category}$		
Observations	19710			19491		
Marginal R^2 / Conditional R^2	0.900 / 0.911			0.079 / 0.156		

Significant effects are marked in bold. Contrasts are sum coded. P-values were obtained via likelihood ratio tests. Conf. Int. Confidence interval.

Table S3. Results of post-hoc tests for significant three-way interaction Age x Condition x Difficulty for accuracy model. P-values are Bonferroni-corrected.

Contrast	Condition	*Odds Ratio*	*SE*	*df*	*Conf. Int (95%)*	*z*	*p*
OA Easy / YA Easy	Categories	0.57	0.12	Inf	0.32 – 0.98	-2.71	**0.040**
OA Easy / OA Difficult	Categories	6.41	1.68	Inf	3.21 – 12.82	7.08	**< 0.001**
OA Easy / YA Difficult	Categories	6.10	1.65	Inf	2.98 – 12.48	6.67	**< 0.001**
YA Easy / OA Difficult	Categories	11.34	3.22	Inf	5.37 – 23.97	8.56	**< 0.001**
YA Easy / YA Difficult	Categories	10.79	2.95	Inf	5.25 – 22.17	8.71	**< 0.001**
OA Difficult / YA Difficult	Categories	0.95	0.11	Inf	0.71 – 1.28	-0.45	1
OA Easy / YA Easy	Counting	0.00	0.00	Inf	0.00 – 0.01	-3.79	**0.001**
OA Easy / OA Difficult	Counting	0.56	0.46	Inf	0.06 – 4.99	-0.71	1
OA Easy / YA Difficult	Counting	0.65	0.53	Inf	0.08 – 5.46	-0.53	1
YA Easy / OA Difficult	Counting	2461403.54	10105894.32	Inf	48.63 – 124584851753.70	3.58	**0.002**
YA Easy / YA Difficult	Counting	2896157.64	11755020.17	Inf	64.76 – 129525179185.02	3.67	**0.001**
OA Difficult / YA Difficult	Counting	1.18	0.61	Inf	0.30 – 4.67	0.31	1

Significant effects are marked in bold. SE standard error; df degrees of freedom; Conf. Int confidence interval.

Table S4. Results of post-hoc tests for significant two-way interaction Age x Condition for response time model. P-values are Bonferroni-corrected.

**Contrast**	***Ratio***	***SE***	***df***	***Conf. Int (95%)***	***z***	***p***
OA Categories / YA Categories	1.05	0.01	Inf	1.03 – 1.07	6.33	**< 0.001**
OA Categories / OA Counting	1.23	0.03	Inf	1.16 – 1.32	8.41	**< 0.001**
OA Categories / YA Counting	1.2	0.03	Inf	1.13 – 1.29	7.28	**< 0.001**
YA Categories / OA Counting	1.18	0.03	Inf	1.1 – 1.26	6.36	**< 0.001**
YA Categories / YA Counting	1.15	0.03	Inf	1.07 – 1.22	5.43	**< 0.001**
OA Counting / YA Counting	0.97	0.01	Inf	0.95 – 0.99	-3.34	0.005

Significant effects are marked in bold. SE standard error; df degrees of freedom; Conf. Int confidence interval.

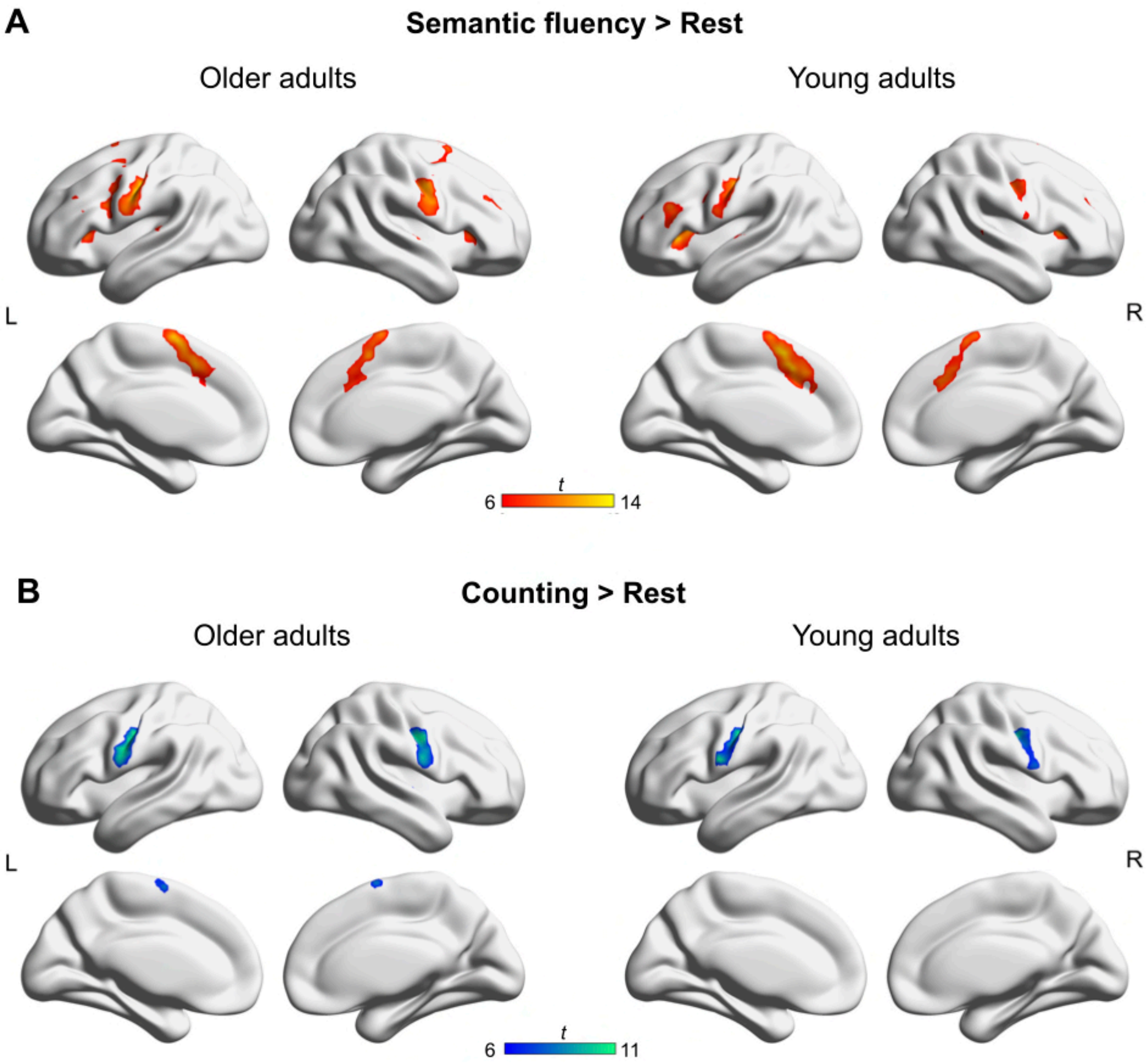

Supplementary Figure S2. Functional MRI results for main effects of tasks from univariate analyses for each age group. Results are FWE-corrected at $p < 0.05$ at peak-level with a minimum cluster size = 20 voxel. Unthresholded statistical maps are available at https://neurovault.org/collections/9072/.

Functional MRI Activation Tables – within-group comparisons

All X, Y, and Z coordinates are in Montreal Neurological Institute (MNI) atlas space. Cluster size (k) is given in mm^3.

Table S5. Older adults: Semantic fluency > Rest.

Anatomical structure	Hemi	*k*	*t*	*x*	*y*	*z*
**Postcentral gyrus**	**L**	**899**	**14.26**	**-46**	**-10**	**35**
Postcentral gyrus	L		12.75	-56	-8	29
Postcentral gyrus	L		12.01	-51	-13	46
Postcentral gyrus	L		11.13	-61	0	24
**Cerebellum**	**L**	**407**	**13.28**	**-34**	**-57**	**-26**
Cerebellum	L		11.9	-16	-62	-15
Cerebellum	L		9.27	-14	-60	-23
Cerebellum	L		9	-16	-75	-20
**Supplementary motor cortex**	**L**	**878**	**13.11**	**-4**	**2**	**62**
Supplementary motor cortex	R		12.98	4	0	68
Supplementary motor cortex	L		11.61	-9	7	57
Superior frontal gyrus	R		11.5	11	5	62
**Postcentral gyrus**	**R**	**370**	**12**	**53**	**-5**	**26**
Precentral gyrus	R		11.77	51	-5	35
Postcentral gyrus	R		8.46	66	-3	18
**Caudate nucleus**	**R**	**86**	**11.63**	**18**	**2**	**24**
Caudate nucleus	R		9.59	16	-8	21
Caudate nucleus	R		8.72	11	0	10
**Cerebellum**	**R**	**571**	**11.58**	**31**	**-65**	**-26**
Cerebellum	R		9.98	28	-67	-56
Cerebellum	R		9.14	21	-72	-53
Cerebellum	R		8.97	36	-55	-50
**Middle frontal gyrus**	**R**	**163**	**11.36**	**36**	**47**	**26**
Middle frontal gyrus	R		9.23	41	35	29
Middle frontal gyrus	R		7.43	26	35	26
Middle frontal gyrus	R		7.28	36	42	18
**Insula**	**L**	**122**	**9.92**	**-31**	**27**	**4**
Insula	L		8.09	-31	15	7
Inferior frontal gyrus, pars orbitalis	L		7.92	-44	37	-9
**Insula**	**R**	**148**	**9.91**	**31**	**27**	**2**
Insula	R		8.77	43	20	2
Inferior frontal gyrus, pars orbitalis	R		6.47	38	32	-6
**Superior temporal gyrus**	**R**	**78**	**9.76**	**63**	**-3**	**2**
Superior temporal gyrus	R		8.24	56	-13	2
Superior temporal gyrus	R		7.49	68	-15	2

Superior temporal gyrus	R		7.06	51	-18	7
**Caudate nucleus**	**L**	**45**	**9.56**	**-16**	**-10**	**24**
Caudate nucleus	L		9.11	-14	-5	16
**Superior parietal lobe**	**L**	**98**	**9.33**	**-19**	**-60**	**48**
Inferior parietal sulcus	L		8.5	-26	-45	38
Inferior parietal lobe	L		7.99	-26	-55	38
Inferior parietal lobe	L		7.77	-41	-40	38
**Superior temporal gyrus**	**L**	**54**	**8.72**	**-51**	**-28**	**10**
Superior temporal gyrus	L		7.68	-66	-23	10
Superior temporal gyrus	L		6.7	-41	-32	10
**Inferior frontal gyrus, pars triangularis**	**L**	**20**	**8.71**	**-36**	**40**	**4**
**Superior temporal gyrus**	**L**	**21**	**8.09**	**-64**	**-10**	**4**

FWE-corrected ($p < 0.05$) at peak level, $k \geq 20$ voxels.

Table S6. Young adults: Semantic fluency > Rest.

Anatomical structure	Hemi	*k*	*t*	*x*	*y*	*z*
**Presupplementary motor cortex**	**L**	**767**	**15.05**	**-4**	**12**	**51**
Supplementary motor cortex	L		13.89	-6	17	43
Supplementary motor cortex	R		12.54	4	7	62
Middle cingulate cortex	R		11.17	11	20	38
**Insula**	**L**	**280**	**13.95**	**-34**	**27**	**2**
Insula	L		11.74	-31	20	7
Inferior frontal gyrus, pars opercularis	L		8.61	-46	10	7
Inferior frontal gyrus, pars opercularis	L		7.01	-49	17	-4
**Cerebellum**	**R**	**683**	**13.82**	**33**	**-55**	**-31**
Cerebellum	R		13.01	43	-60	-28
Cerebellum	R		11.51	33	-62	-50
Cerebellum	R		11.24	26	-67	-48
**Postcentral gyrus**	**L**	**331**	**13.74**	**-49**	**-15**	**40**
Postcentral gyrus	L		10.89	-56	-13	46
Postcentral gyrus	L		9.93	-61	0	21
Precentral gyrus	L		8.04	-54	0	46
**Cerebellum**	**L**	**377**	**13.18**	**-26**	**-60**	**-26**
Cerebellum	L		12.1	-46	-62	-28
**Insula**	**R**	**130**	**12.01**	**33**	**22**	**7**
Inferior frontal gyrus, pars opercularis	R		9.4	46	15	4
Insula	R		9.05	41	20	-1
**Cerebellum**	**R**	**40**	**10.86**	**1**	**-47**	**-23**

**Cerebellum**	**L**	**68**	**10.41**	**-36**	**-60**	**-50**
**Precentral gyrus**	**R**	**208**	**10.23**	**56**	**-3**	**46**
Precentral gyrus	R		10.09	46	-10	38
Postcentral gyrus	R		8.94	56	-5	35
Rolandic operculum	R		7.71	61	-3	16
**Inferior frontal gyrus, pars triangularis**	**L**	**208**	**9.99**	**-44**	**32**	**24**
Inferior frontal gyrus, pars triangularis	L		8.29	-51	30	21
Inferior frontal gyrus, pars triangularis	L		8.13	-39	35	7
Inferior frontal gyrus, pars triangularis	L		7.96	-51	35	10
**Thalamus**	**L**	**57**	**9.31**	**-11**	**-5**	**13**
Caudate nucleus	L		9.2	-16	-3	21
**Inferior frontal gyrus, pars opercularis**	**L**	**98**	**8.33**	**-39**	**2**	**26**
Precentral gyrus	L		7.6	-46	10	32
Precentral gyrus	L		7.16	-41	0	38
Inferior frontal gyrus, pars triangularis	L		6.5	-46	15	24
**Middle frontal gyrus**	**R**	**81**	**8.1**	**33**	**47**	**32**
Middle frontal gyrus	R		7.08	31	47	24
**Caudate nucleus**	**R**	**43**	**8.09**	**18**	**5**	**21**
Caudate nucleus	R		7.75	16	-3	24
Caudate nucleus	R		7.73	18	12	16
**Middle frontal gyrus**	**L**	**26**	**7.57**	**-34**	**55**	**21**
**Superior temporal gyrus**	**R**	**21**	**7.5**	**66**	**-30**	**7**
Superior temporal gyrus	R		6.7	56	-30	4
**Superior temporal gyrus**	**L**	**20**	**6.74**	**-59**	**-15**	**4**

FWE-corrected ($p < 0.05$) at peak level, $k \geq 20$ voxels.

Table S7. Older adults: Counting > Rest.

**Anatomical structure**	**Hemi**	***k***	***t***	***x***	***y***	***z***
**Postcentral gyrus**	**L**	**347**	**11.69**	**-46**	**-13**	**38**
Postcentral gyrus	L		11.56	-61	-3	24
Postcentral gyrus	L		11.52	-51	-13	46
Postcentral gyrus	L		11.05	-56	-8	29
**Postcentral gyrus**	**R**	**342**	**11.43**	**48**	**-8**	**35**
Postcentral gyrus	R		10.71	53	-3	24
Postcentral gyrus	R		9.39	63	-3	18
**Supplementary motor cortex**	**L**	**57**	**10.16**	**-4**	**-5**	**70**
**Supplementary motor cortex**	**R**	**59**	**9.96**	**4**	**0**	**68**

**Cerebellum**	**L**	**52**	**9.35**	**-29**	**-62**	**-23**
Cerebellum	L		7.84	-16	-65	-18
**Superior temporal gyrus**	**R**	**49**	**8.44**	**66**	**-10**	**2**
Superior temporal gyrus	R		7.71	63	2	-1
Superior temporal gyrus	R		6.96	68	-18	4
Superior temporal gyrus	R		6.76	53	-15	4
**Superior temporal gyrus**	**L**	**46**	**8.4**	**-46**	**-42**	**21**
Superior temporal gyrus	L		6.54	-46	-37	13
Superior temporal gyrus	L		6.19	-51	-28	10
**Cerebellum**	**R**	**26**	**6.96**	**21**	**-60**	**-23**
Cerebellum	R		6.9	11	-60	-23

FWE-corrected ($p < 0.05$) at peak level, $k \geq 20$ voxels.

Table S8. Young adults: Counting > Rest.

Anatomical structure	Hemi	*k*	*t*	*x*	*y*	*z*
**Postcentral gyrus**	**L**	**317**	**11.47**	**-61**	**0**	**21**
Postcentral gyrus	L		11.3	-49	-15	40
Postcentral gyrus	L		9.28	-56	-13	46
Postcentral gyrus	L		6.99	-59	-8	16
**Precentral gyrus**	**R**	**247**	**10.42**	**46**	**-10**	**38**
Precentral gyrus	R		9.44	56	-3	46
Rolandic operculum	R		9.35	61	2	16
Postcentral gyrus	R		8.25	56	-8	35
**Cerebellum**	**R**	**37**	**8.3**	**13**	**-60**	**-20**
**Cerebellum**	**L**	**25**	**7.37**	**-16**	**-60**	**-23**

FWE-corrected ($p < 0.05$) at peak level, $k \geq 20$ voxels.

Table S9. Older adults: Semantic fluency > Counting.

Anatomical structure	Hemi	*k*	*t*	*x*	*y*	*z*
**Cerebellum**	**L**	**1844**	**12.99**	**-36**	**-62**	**-26**
Cerebellum	R		12.59	28	-62	-26
Cerebellum	L		12.03	-29	-67	-26
Cerebellum	R		11.08	6	-80	-31
**Middle frontal gyrus**	**L**	**578**	**12.9**	**-44**	**5**	**35**
Inferior frontal gyrus, pars opercularis	L		11.2	-41	15	21
Precentral gyrus	L		10.7	-39	2	24
Middle frontal gyrus	L		8.94	-46	7	46
**Superior frontal gyrus (preSMA)**	**L**	**661**	**12.02**	**-9**	**15**	**51**
Presupplementary motor cortex	L		11.62	-9	20	43
Superior frontal gyrus	L		10.34	-1	10	60

Superior frontal gyrus	R		9.34	8	15	48
**Insula**	**L**	**269**	**10.98**	**-31**	**25**	**4**
Caudate nucleus	L		10.84	-16	0	16
Caudate nucleus	L		9.52	-16	-10	21
Superior frontal gyrus	L		8.74	-19	10	4
**Insula**	**R**	**113**	**10.95**	**31**	**27**	**2**
Inferior frontal gyrus. pars triangularis	R		7.25	48	22	-4
Frontal operculum	R		7.08	43	20	4
**Caudate nucleus**	**R**	**106**	**9.96**	**18**	**15**	**18**
Caudate nucleus	R		9.53	18	-8	21
Caudate nucleus	R		8.67	16	0	18
Thalamus	R		8.22	11	0	10
**Middle frontal gyrus**	**R**	**36**	**9.79**	**43**	**35**	**32**
**Superior frontal gyrus**	**L**	**100**	**9.12**	**-21**	**12**	**54**
Middle frontal gyrus	L		6.82	-21	-3	60
Middle frontal gyrus	L		6.57	-24	20	51
**Intracalcarine cortex**	**R**	**43**	**9.01**	**18**	**-80**	**7**
Occipital pole	R		7.93	13	-95	10
Intracalcarine cortex	R		6.77	13	-77	16
**Angular gyrus**	**L**	**27**	**8.1**	**-34**	**-72**	**43**
**Middle frontal gyrus**	**L**	**24**	**8.07**	**-34**	**0**	**57**
**Superior parietal lobe**	**L**	**61**	**7.65**	**-14**	**-65**	**51**
Superior parietal lobe	L		7.31	-21	-65	60
Angular gyrus	L		6.65	-29	-62	46
**Intracalcarine cortex**	**L**	**71**	**7.52**	**-11**	**-72**	**10**
Intracalcarine cortex	L		7.24	-6	-87	2
Intracalcarine cortex	L		6.96	-4	-82	10
**Middle frontal gyrus**	**R**	**21**	**7.39**	**23**	**60**	**-4**
Middle frontal gyrus	R		7.05	31	57	-9
**Thalamus**	**L**	**21**	**6.99**	**-4**	**-5**	**10**

FWE-corrected ($p < 0.05$) at peak level, $k \geq 20$ voxels.

Table S10. Young adults: Semantic fluency > Counting.

**Anatomical structure**	**Hemi**	***k***	***t***	***x***	***y***	***z***
**Insula**	**L**	**3112**	**20.03**	**-31**	**25**	**2**
Presupplementary motor cortex	L		16.85	-4	25	40
Presupplementary motor cortex	L		16.33	-6	12	51
Presupplementary motor cortex	R		14.72	13	27	29
**Cerebellum**	**R**	**2970**	**19.11**	**33**	**-57**	**-31**
Cerebellum	R		16.12	31	-65	-28
Cerebellum	R		13.89	28	-70	-50
Cerebellum	R		12.66	41	-60	-28

**Anterior cingulate gyrus**	**L**	**90**	**14.15**	**-4**	**2**	**29**
Anterior cingulate gyrus	L		9.12	-1	12	24
Anterior cingulate gyrus	R		8.89	6	7	26
**Insula**	**R**	**315**	**13.32**	**31**	**27**	**2**
Insula	R		12.64	38	20	-4
**Caudate nucleus**	**L**	**235**	**12.81**	**-9**	**5**	**2**
Caudate nucleus	L		12.02	-16	-3	21
Thalamus	L		11.32	-11	-5	13
Caudate nucleus	L		10.63	-16	7	16
**Brain stem**	**L**	**296**	**12.46**	**-6**	**-23**	**-18**
Thalamus	L		11.94	-9	-18	16
Thalamus	L		11.85	-4	-23	10
Thalamus	L		10.36	-4	-13	10
**Superior parietal lobe**	**L**	**224**	**10.49**	**-29**	**-65**	**51**
Angular gyrus	L		10.21	-29	-72	43
Inferior parietal lobe	L		8.84	-34	-57	40
Middle occipital gyrus	L		6.47	-31	-80	38
**Caudate nucleus**	**R**	**215**	**10.45**	**18**	**10**	**18**
Caudate nucleus	R		10.38	8	7	2
Caudate nucleus	R		9.57	13	7	10
Caudate nucleus	R		9.16	18	-3	21
**Superior temporal gyrus**	**L**	**54**	**9.62**	**-61**	**-30**	**7**
Planum temporale	L		6.97	-61	-15	4
**Middle frontal gyrus**	**R**	**176**	**8.3**	**36**	**42**	**32**
Middle frontal gyrus	R		7.56	31	55	26
Middle frontal gyrus	R		7.53	33	37	21
Middle frontal gyrus	R		7.17	41	35	40

FWE-corrected ($p < 0.05$) at peak level, $k \geq 20$ voxels.

Table S11. Older adults: Counting > Semantic fluency.

Anatomical structure	Hemi	*k*	*t*	*x*	*y*	*z*
**Temporal pole**	**R**	**30**	**9.33**	**51**	**12**	**-31**
**Precuneus**	**R**	**45**	**7.72**	**6**	**-52**	**38**

FWE-corrected ($p < 0.05$) at peak level, $k \geq 20$ voxels.

Table S12. Young adults: Counting > Semantic fluency (FWE-corrected at peak level).

Anatomical structure	Hemi	*k*	*t*	*x*	*y*	*z*
**Temporal pole**	**R**	**75**	**11.02**	**51**	**10**	**-31**
Temporal pole	R		6.93	43	20	-28
**Precuneus**	**R**	**312**	**9.7**	**8**	**-65**	**29**
Precuneus	R		9.59	11	-52	35
Precuneus	L		9.05	-9	-52	35

**Insula**	**L**	**46**	**8.62**	**-41**	**-8**	**-1**
Insula	L		7.24	-36	-18	18
Insula	L		7.02	-39	-15	2
**Insula**	**R**	**62**	**8.48**	**36**	**-15**	**4**
Insula	R		7.66	41	0	-6
Insula	R		7.1	38	-15	21
**Middle temporal gyrus**	**L**	**27**	**8.27**	**-56**	**2**	**-20**
**Rolandic operculum**	**R**	**22**	**8.01**	**53**	**0**	**10**
**Posterior cingulate cortex**	**L**	**40**	**7.9**	**-6**	**-30**	**46**
Precentral gyrus	L		6.77	-6	-25	54
**Precentral gyrus**	**R**	**28**	**7.42**	**1**	**-15**	**62**
Precentral gyrus	L		6.91	-4	-23	70

FWE-corrected ($p < 0.05$) at peak level, $k \geq 20$ voxels.

Table S13. Young adults: Counting > Semantic fluency ($p < 0.001$ uncorr., FWE-corrected $p < 0.05$ at cluster level).

Anatomical structure	Hemi	*k*	*t*	*x*	*y*	*z*
**Temporal pole**	**R**	**281**	**11.02**	**51**	**10**	**-31**
Temporal pole	R		6.93	43	20	-28
Middle temporal gyrus	R		5.93	61	-8	-12
Superior temporal gyrus	R		4.35	48	-10	-15
**Precuneus**	**R**	**3620**	**9.7**	**8**	**-65**	**29**
Precuneus	R		9.59	11	-52	35
Precuneus	L		9.05	-9	-52	35
Insula	R		8.48	36	-15	4
Central operculum	R		8.01	53	0	10
**Insula**	**L**	**438**	**8.62**	**-41**	**-8**	**-1**
Insula	L		7.24	-36	-18	18
Insula	L		7.02	-39	-15	2
Precentral gyrus	L		6.99	-59	2	10
Insula	L		5.22	-41	-3	-12
**Middle temporal gyrus**	**L**	**151**	**8.27**	**-56**	**2**	**-20**
Temporal pole	L		7.06	-54	10	-31
Temporal pole	L		4.5	-44	20	-31
Temporal pole	L		3.99	-41	7	-23
**Anterior cingulate cortex**	**L**	**96**	**7.36**	**-6**	**27**	**-6**
Anterior cingulate cortex	L		4.09	-6	42	-4
**Angular gyrus**	**L**	**281**	**7.11**	**-54**	**-62**	**35**
Angular gyrus	L		5.46	-41	-60	26
Middle temporal gyrus	L		5.05	-46	-62	18
Angular gyrus	L		4.46	-46	-75	35
Angular gyrus	L		4.03	-49	-67	43
**Angular gyrus**	**R**	**389**	**7.02**	**51**	**-57**	**26**

Angular gyrus	R	4.6	46	-65	48
Angular gyrus	R	4.16	43	-72	35
Lateral occipital cortex	R	4.1	46	-77	26
Lateral occipital cortex	R	3.55	56	-62	7

FWE-corrected ($p < 0.05$) at cluster level, $p < 0.001$ uncorr. at peak level.

Table S14. Results for linear mixed-effects model for parameter estimates from fMRI main effects.

		**Beta weights**	
Coefficient	*Estimates*	*Conf. Int (95%)*	*p*
Intercept	-0.39	-0.54 – -0.24	**< 0.001**
Network	1.07	0.95 – 1.19	**< 0.001**
Age	0.34	0.21 – 0.46	**< 0.001**
Condition	0.49	0.37 – 0.61	**< 0.001**
Network * Age	-0.02	-0.15 – 0.10	0.704
Network * Condition	1.14	1.02 – 1.26	**< 0.001**
Age * Condition	-0.02	-0.14 – 0.10	0.762
Network * Age * Condition	-0.27	-0.39 – -0.15	**< 0.001**
**Random Effects**			
σ^2	0.91		
$\tau_{00\ Subj}$	0.06		
ICC	0.06		
N_{Subj}	58		
Observations	232		
Marginal R^2 / Conditional R^2	0.751 / 0.766		

Significant effects are marked in bold. Contrasts are sum coded. P-values were obtained via likelihood ratio tests. Conf. Int. Confidence interval.

Functional MRI Results – within-group comparisons

Table S15. Results for post-hoc tests for significant three-way interaction Network x Age x Contrast for parameter estimates model. P-values are Bonferroni-corrected.

Contrast	fMRI contrast	*Estimate*	*SE*	*df*	*Conf. Int (95%)*	*t*	*p*
OA MDN - YA MDN	SF > rest	0.04	0.25	196.93	-0.63 – 0.71	0.17	1
OA MDN - OA DMN	SF > rest	3.83	0.25	195.3	3.15 – 4.51	15.05	**< 0.001**
OA MDN - YA DMN	SF > rest	5.05	0.25	196.93	4.38 – 5.72	20.18	**< 0.001**
YA MDN - OA DMN	SF > rest	3.79	0.25	196.93	3.12 – 4.45	15.12	**< 0.001**
YA MDN - YA DMN	SF > rest	5.01	0.25	195.3	4.35 – 5.66	20.38	**< 0.001**
OA DMN - YA DMN	SF > rest	1.22	0.25	196.93	0.56 – 1.89	4.89	**< 0.001**
OA MDN - YA MDN	Count > rest	1.20	0.25	196.93	0.54 – 1.87	4.81	**< 0.001**
OA MDN - OA DMN	Count > rest	0.35	0.25	195.3	-0.33 – 1.03	1.38	1
OA MDN - YA DMN	Count > rest	0.57	0.25	196.93	-0.10 – 1.24	2.27	0.146
YA MDN - OA DMN	Count > rest	-0.85	0.25	196.93	-1.52 – -0.19	-3.41	**0.005**
YA MDN - YA DMN	Count > rest	-0.64	0.25	195.3	-1.29 – 0.02	-2.59	0.062
OA DMN - YA DMN	Count > rest	0.22	0.25	196.93	-0.45 – 0.89	0.87	1

Significant effects are marked in bold. SF semantic fluency; Count counting; MDN multiple-demand network; DMN default mode network; SE standard error; df degrees of freedom; Conf. Int confidence interval.

Table S 16. Young adults: Easy > Difficult semantic categories.

Anatomical structure	Hemi	*k*	*t*	*x*	*y*	*z*
**Middle frontal gyrus**	**R**	**20**	**7.24**	**26**	**2**	**51**
Middle frontal gyrus	R		6.49	31	12	51

FWE-corrected ($p < 0.05$) at peak level, $k \geq 20$ voxels.

Functional Connectivity Results

Activation Tables for Psychophysiological Interactions (PPI)

All individual seeds for PPIs were thresholded at $p < 0.01$. All reported results are for the contrast Semantic fluency > Counting and are FWE-corrected at $p < 0.05$ at peak level (k ≥ 20 voxels). All X, Y, and Z coordinates are in Montreal Neurological Institute (MNI) atlas space. Cluster size (k) is given in mm^3.

Table S17. PPI seed: Pre-supplementary Motor Area [-6 12 51].

Anatomical structure	Hemi	*k*	*t*	*x*	*y*	*z*
**Older adults**						
No significant clusters above threshold.						
**Young adults**						
**Caudate nucleus**	**L**	**92**	**9.89**	**-14**	**10**	**4**
Caudate nucleus	L		8.68	-16	20	4
Caudate nucleus	L		6.18	-11	7	13
**Caudate nucleus**	**R**	**76**	**9.19**	**8**	**12**	**2**
Caudate nucleus	R		7.72	18	22	-4
Putamen	R		7.30	18	12	-1
Caudate nucleus	R		6.93	18	25	4
**Precuneus**	**L**	**125**	**8.31**	**-6**	**-52**	**16**
Posterior cingulate cortex	L		7.60	-4	-55	26
Precuneus	L		7.44	-11	-55	7
**Thalamus**	**L**	**35**	**7.30**	**-1**	**-13**	**7**
Thalamus	R		7.29	4	-20	10
Thalamus	L		6.50	-9	-25	13

Table S18. PPI seed: Left Insula [-31 25 2].

Anatomical structure	Hemi	*k*	*t*	x	y	z
**Older adults**						
No significant clusters above threshold.						
**Young adults**						
**Caudate nucleus**	**R**	**36**	**10.09**	**8**	**12**	**2**
**Caudate nucleus**	**L**	**34**	**7.04**	**-14**	**12**	**4**
Caudate nucleus	L		6.33	-16	22	2
**Precuneus**	**L**	**31**	**6.88**	**-9**	**-60**	**7**
Precuneus	L		6.75	-6	-52	16

Table S19. PPI seed: Right Insula [31 27 2].

Anatomical structure	Hemi	*k*	*t*	*x*	*y*	*z*
**Older adults**						

**Precuneus**	**R**	**29**	**8.92**	**1**	**-60**	**26**
Posterior cingulate gyrus	L		8.56	-1	-45	26
**Inferior frontal gyrus, pars orbitalis**	**L**	**20**	**7.39**	**-31**	**35**	**-15**
Inferior frontal gyrus, pars orbitalis	L		6.46	-44	30	-12
**Young adults**						
No significant clusters above threshold.						

Table S20. PPI seed: Right Temporal Pole [48 15 -31].

Anatomical structure	Hemi	*k*	*t*	*x*	*y*	*z*
**Older adults**						
**Inferior frontal gyrus, pars opercularis**	**R**	**24**	**7.33**	**51**	**17**	**-1**
Insula	R		7.02	41	27	-1
Insula	R		6.31	41	12	-1
**Young adults**						
**Inferior frontal gyrus, pars opercularis**	**R**	**47**	**8.48**	**43**	**12**	**21**
Inferior frontal gyrus, pars opercularis	R		7.08	53	12	18
**Superior frontal gyrus**	**R**	**46**	**8.28**	**18**	**2**	**65**
Middle frontal gyrus	R		6.94	31	-3	62
Superior frontal gyrus	R		6.44	21	12	65
**Insula**	**R**	**75**	**7.22**	**43**	**15**	**2**
Frontal operculum	R		6.76	33	25	7
Frontal operculum	R		6.53	36	15	10
**Supramarginal gyrus**	**R**	**23**	**7.21**	**58**	**-32**	**48**

Table S21. PPI seed: Right Precuneus [8 -65 29].

Anatomical structure	Hemi	*k*	*t*	*x*	*y*	*z*
**Older adults**						
**Insula**	**R**	**295**	**9.27**	**33**	**22**	**10**
Inferior frontal gyrus, pars triangularis	R		9.27	53	25	10
Insula	R		8.97	41	25	-6
Inferior frontal gyrus, pars triangularis	R		8.79	46	25	4
**Supramarginal gyrus**	**R**	**410**	**9.20**	**53**	**-40**	**46**
Angular gyrus	R		9.18	56	-45	32
Supramarginal gyrus	R		8.91	63	-42	35
Angular gyrus	R		8.72	56	-47	48
**Middle frontal gyrus**	**R**	**125**	**8.78**	**33**	**47**	**32**
Middle frontal gyrus	R		8.04	41	42	29
Middle frontal gyrus	R		7.39	46	45	21
**Supramarginal gyrus**	**L**	**42**	**8.77**	**-61**	**-47**	**32**

Supramarginal gyrus	L		6.89	-54	-50	35
**Inferior frontal gyrus, pars orbitalis**	**R**	**61**	**8.4**	**48**	**45**	**-6**
Inferior frontal gyrus, pars triangularis	R		8.03	51	37	-1
Inferior frontal gyrus, pars triangularis	R		7.78	48	40	7
Inferior frontal gyrus, pars triangularis	R		7.38	41	42	-1
**Presupplementary motor area**	**R**	**35**	**8.06**	**4**	**7**	**60**
Presupplementary motor area	R		7.44	6	10	68
**Inferior frontal gyrus, pars triangularis**	**L**	**70**	**8.02**	**-41**	**17**	**7**
Inferior frontal gyrus, pars triangularis	L		7.85	-36	30	4
Inferior frontal gyrus, pars opercularis	L		6.84	-46	10	7
**Superior temporal gyrus**	**R**	**32**	**7.86**	**53**	**-15**	**-4**
Middle temporal gyrus	R		7.03	56	-30	-1
Superior temporal gyrus	R		6.77	63	-20	-1
**Precentral gyrus**	**R**	**93**	**7.85**	**46**	**7**	**35**
Middle frontal gyrus	R		6.89	41	10	46
Middle frontal gyrus	R		6.88	41	15	29
Precentral gyrus	R		6.82	43	5	26
**Inferior frontal gyrus, pars opercularis**	**R**	**21**	**7.77**	**56**	**15**	**24**
**Angular gyrus**	**L**	**39**	**7.61**	**-51**	**-52**	**48**
Supramarginal gyrus	L		7.19	-59	-47	43
**Lateral occipital cortex**	**R**	**29**	**7.15**	**33**	**-67**	**29**
Lateral occipital cortex	R		6.78	26	-77	26
**Young adults**						
**Supramarginal gyrus**	**R**	**1300**	**9.69**	**61**	**-45**	**26**
Angular gyrus	R		8.29	63	-47	18
Supramarginal gyrus	R		8.28	51	-42	13
Supramarginal gyrus	R		8.17	58	-32	43
**Superior frontal gyrus**	**R**	**21**	**9.35**	**8**	**30**	**54**
**Superior frontal gyrus**	**R**	**185**	**9.03**	**11**	**5**	**62**
Superior frontal gyrus	R		8.40	16	12	65
Superior frontal gyrus	R		7.35	11	-10	68
Superior frontal gyrus	R		6.78	18	-3	73
**Insula**	**L**	**194**	**8.80**	**-44**	**10**	**-4**
Insula	L		7.86	-46	2	4
Insula	L		7.81	-34	2	0
Insula	L		6.91	-44	22	-6
**Precentral gyrus**	**R**	**79**	**8.36**	**46**	**-3**	**48**
Middle frontal gyrus	R		6.85	43	-3	57
Precentral gyrus	R		6.53	51	2	40
Precentral gyrus	R		6.49	33	-8	48
**Anterior cingulate cortex, dorsal part**	**R**	**147**	**7.97**	**11**	**17**	**35**

Anterior cingulate cortex, dorsal part	L		7.14	-1	5	43
Anterior cingulate cortex	L		6.79	-4	25	24
Anterior cingulate cortex, dorsal part	R		6.00	4	-5	40
**Middle temporal gyrus**	**R**	**161**	**7.92**	**48**	**-60**	**13**
Middle temporal gyrus	R		7.44	56	-52	2
Middle temporal gyrus	R		6.88	56	-57	10
Middle temporal gyrus	R		6.34	43	-67	-1
**Angular gyrus**	**L**	**201**	**7.84**	**-61**	**-50**	**35**
Supramarginal gyrus	L		7.80	-64	-47	26
Supramarginal gyrus	L		7.40	-64	-40	32
Central operculum	L		6.85	-59	-23	16
**Posterior cingulate cortex**	**R**	**187**	**7.60**	**8**	**-30**	**46**
Precuneus	R		7.35	6	-42	51
Precentral gyrus	R		7.35	6	-18	48
Precuneus	R		7.15	8	-57	62
**Posterior cingulate cortex**	**L**	**24**	**7.59**	**-1**	**-25**	**26**
Posterior cingulate cortex	R		6.86	6	-28	29
**Superior occipital gyrus**	**L**	**24**	**7.39**	**-16**	**-77**	**43**
**Lateral occipital cortex**	**L**	**38**	**7.34**	**-31**	**-82**	**16**
Lateral occipital cortex	L		6.37	-29	-92	18
**Cuneus**	**R**	**31**	**7.32**	**18**	**-82**	**26**
**Cuneus**	**R**	**58**	**7.30**	**13**	**-75**	**26**
Lateral occipital cortex	R		6.51	13	-75	46
Cuneus	R		6.23	13	-75	35
Cuneus	R		6.0	8	-80	40
**Lateral occipital cortex**	**R**	**61**	**7.30**	**31**	**-75**	**24**
**Calcarine gyrus**	**R**	**50**	**7.11**	**1**	**-70**	**13**
Lingual gyrus	R		6.44	4	-80	2
Intracalcarine cortex	L		6.38	-6	-75	18
**Postcentral gyrus**	**L**	**20**	**6.74**	**-9**	**-47**	**57**
Precentral gyrus	L		6.33	-14	-37	43
**Fusiform gyrus**	**R**	**24**	**6.67**	**36**	**-67**	**-15**
Fusiform gyrus	R		6.66	28	-72	-12
**Middle frontal gyrus**	**R**	**23**	**6.51**	**31**	**40**	**26**
Middle frontal gyrus	R		6.22	41	42	29

Result Tables for functional connectivity

Table S22. Results for linear model for within and between network connectivity.

PPI variable	FC	IV	*b*	*SE*	*t*	*p*
Semantic fluency > Counting	Within MDN	Intercept	0.05	0.02	2.70	**0.009**
		Age	0.005	0.03	0.18	0.85
	Within DMN	Intercept	-0.002	0.02	-0.07	0.95
		Age	0.02	0.03	0.50	0.63
	Between MDN and DMN	Intercept	0.10	0.02	4.47	**< 0.001**
		Age	0.04	0.03	1.25	0.22

Significant effects are marked in bold: p < *Meff*-corrected α of 0.018; FC Functional connectivity; IV Independent variable; MDN Multiple-demand network; DMN Default-mode network.

Table S23. Results for generalized linear mixed models for within- and between-network functional connectivity effects, age, and condition on accuracy and response time.

	**Accuracy**			**Response time**		
Coefficient	*Log-Odds*	*Conf. Int (95%)*	*p*	*Estimates*	*Conf. Int (95%)*	*p*
Intercept	3.09	2.48 – 3.70	**< 0.001**	6.53	6.48 – 6.58	**< 0.001**
Within-MDN FC	0.22	-1.41 – 1.84	0.592	-0.13	-0.28 – 0.03	0.493
Within-DMN FC	-0.82	-2.42 – 0.79	0.302	-0.35	-0.53 – -0.18	**< 0.001**
Between-network FC	0.56	-1.22 – 2.34	0.556	0.53	0.36 – 0.71	**< 0.001**
Age	-0.17	-0.29 – -0.01	0.11	-0.01	-0.02 – 0.01	**< 0.001**
Education	-0.15	-0.29 – -0.01	**0.028**	-0.01	-0.02 – 0.01	0.061
Within-MDN FC * Age	1.15	-1.93 – 4.23	0.469	0.94	0.60 – 1.28	**< 0.001**
Within-DMN FC * Age	2.06	-0.77 – 4.90	0.157	0.90	0.62 – 1.19	**< 0.001**
Between-network FC * Age	-2.41	-5.42 – 0.60	0.119	-0.79	-1.10 – -0.47	**< 0.001**

Random Effects

σ^2	3.29	0.13
τ_{00}	0.22 $_{Subj}$	0.01 $_{Subj}$
	1.71 $_{Category}$	0.00 $_{Category}$
ICC	0.37	0.12
N	58 $_{Subj}$	58 $_{Subj}$
	20 $_{Category}$	20 $_{Category}$
Observations	9837	9675
Marginal R^2 / Conditional R^2	0.002 / 0.371	0.027 / 0.143

Significant effects are marked in bold. Contrasts are sum coded. P-values were obtained via likelihood ratio tests. Conf. Int. Confidence interval.

Table S24. Results of post-hoc tests for two-way interactions Age x Connectivity measure for response time model. P-values are Bonferroni-corrected.

**Contrast**	**FC**	***Estimate***	***SE***	***df***	***Conf. Int (95%)***	***z***	***p***
OA – YA	Within MDN	636	118	Inf	405 – 867	5.39	**< 0.001**
	Within DMN	604	100	Inf	407 – 800	6	**< 0.001**
	Between MDN and DMN	-516	110	Inf	-732 – -301	-4.69	**< 0.001**

Significant effects are marked in bold. FC functional connectivity; SE standard error; df degrees of freedom; Conf. Int confidence intervals.
